# Resuscitation after birth and beyond in the neonatal intensive care unit: NRP or PALS?

**DOI:** 10.1038/s41372-025-02348-4

**Published:** 2025-07-29

**Authors:** Srinivasan Mani, Mausma Bawa, Nishant Srinivasan, Valerie Elberson, Anne Marie Reynolds, Jeremy Killion, Mary Emborsky, Bree Kramer, Shahzad Waheed, Mary Kasu, Arun Prasath, Mary Miller, Munmun Rawat, Praveen Chandrasekharan

**Affiliations:** 1 Jacobs School of Medicine, Department of Pediatrics, SUNY at Buffalo, Buffalo, NY USA; 2 Division of Neonatology, SUNY at Buffalo, Buffalo, NY USA; 3 University of Illinois, Chicago, IL USA; 4 Division of Pediatric Emergency Room, SUNY at Buffalo, Buffalo, NY USA; 5 Division of Pediatric Intensive Care Unit, SUNY at Buffalo, Buffalo, NY USA; 6 University of Maryland, College Park, MA USA; 7 UT Southwestern Medical Center, Dallas, TX USA; 8 Kaleida Health, Division of Neonatology, Buffalo, NY USA

**Keywords:** Medical research, Developing world

## Abstract

Newborns requiring resuscitation present a unique challenge compared to pediatric and adult patients due to the physiological differences at birth. This paper explores the distinction between the Neonatal Resuscitation Program (NRP) and Pediatric Advanced Life Support (PALS) in neonatal intensive care units (NICUs), with a focus on the optimal approaches for resuscitation in newborns. Through clinical studies and case scenario analysis, the paper underscores the importance of ventilation, tailored algorithms, and collaborative resuscitation efforts in NICU settings, focusing on respiratory versus cardiac causes. It evaluates the necessity of NRP over PALS, the resuscitation techniques, and the impact of combining the two protocols. A perspective on the challenges and costs of implementing such protocols has also been discussed.

## Introduction

Birth asphyxia is the most common cause necessitating cardiopulmonary resuscitation (CPR) at birth [1]. The uniqueness of newborn resuscitation is due to the physiological cardiopulmonary transition occurring at birth [2]. The lungs, which are fluid-filled, take over as an organ of gas exchange from the placenta while transitioning from fetal to neonatal life. Any event that disrupts this process can lead to fetal hypoxemia and hypercarbia, resulting in acidosis. This could subsequently impair perfusion and, if not addressed by appropriate resuscitative efforts, lead to bradycardia and circulatory failure [3, 4]. In neonates, the focus is primarily on the airway, breathing followed by circulation, in contrast to resuscitation in cardiopulmonary arrest secondary to primary heart disease, where circulation is emphasized. In infants >28 d and patients until 18 y age, the etiology for resuscitation may similarly differ [5]. The resuscitation outside the newborn period focuses on circulation, airway, and breathing to maintain circulation until specific treatments like defibrillation restore the heart’s function [6].

Newborns born outside the hospital who present to the emergency room (ER) or neonates (<28 days old) admitted to a tertiary intensive care unit and require resuscitation either follow a Neonatal Resuscitation Program (NRP) or Pediatric Advanced Life Support (PALS). The optimization of CPR approaches for hospitalized neonates and infants is a critical concern due to the high incidence of cardiopulmonary instability and arrest in this population. Notably, CPR with chest compressions (CC) is required in 0.25% to 1% of neonatal intensive care unit (NICU) admissions, 1.4% of pediatric intensive care unit (PICU) admissions, and 3.1% of pediatric cardiac ICU (PCICU) admissions, predominantly affecting patients under one year of age. The incidence of CPR in the NICU is significantly higher than that at birth, where CPR is required in only 0.06% to 0.12% of cases [7–10]. Resuscitation practices differ based on the patient’s age, arrest physiology, and care location, with distinct neonatal and pediatric resuscitation guidelines.

## The need for clear guidelines on transitioning from Neonatal to Pediatric Resuscitation Protocols

Neonatal guidelines emphasize effective lung ventilation, while pediatric guidelines focus on addressing the etiology of arrest and ensuring effective cardiac compressions. These guidelines also vary regarding CPR sequence, chest compression-to-ventilation ratios, coordination of breaths with compressions, and medications. Despite the recognized need for transitioning from neonatal to pediatric resuscitation protocols as infants age, there is no established guidance on timing and under what circumstances to make this shift [11]. The lack of evidence-based recommendations results in inconsistent resuscitation protocol application, leading to potential practice variability. This inconsistency is concerning as it may adversely affect the quality of care and contribute to suboptimal resuscitation outcomes. Therefore, clear evidence-based guidelines must be established to inform patient care providers of the transition from neonatal to pediatric resuscitation protocols for hospitalized neonates and infants to minimize practice variability and potentially improve outcomes.

## Discussion

### Below are clinical scenarios with considerations and questions on approaches that could happen in a children’s hospital

#### Protocol application and transition in neonatal resuscitation scenarios

Scenario 1: A 30-minute-old term neonate delivered at home presented to the emergency room (ER) in critical condition—pale, mottled, with poor respiratory efforts and no detectable heart rate.

In this scenario, using PALS, followed by a transition to NRP, is a reasonable approach. In an emergency scenario, immediate resuscitation is paramount to ensure survival. PALS guidelines are often necessary, especially if a neonatal provider is not immediately available. PALS emphasizes immediate CC and ventilation strategies that are applicable to pediatric resuscitation, addressing common causes of cardiac arrest. However, once a neonatal provider arrives, it is crucial to transition to NRP, since NRP is specifically designed for newborns.

NRP incorporates strategies that focus on neonates’ unique physiological characteristics, such as effective ventilation, thermoregulation, and careful oxygenation management. These aspects are critical for optimal neonatal care, particularly in the early minutes after birth, when physiological immaturity requires specialized attention.

This scenario illustrates the current gap in evidence on protocols, where initial management may lean on PALS in the absence of specialized care providers. While PALS provides a general framework for pediatric resuscitation, NRP addresses the nuances of neonatal care, ensuring the most appropriate interventions for newborns. Therefore, the initial use of PALS in this context is acceptable to provide immediate life-saving interventions, but the subsequent transition to NRP ensures that the resuscitation process is tailored to the newborn’s specific needs.

The question of whether this approach is correct hinges on the timing of provider availability and the nature of the resuscitation. The initial use of PALS may serve as a stopgap measure, but transitioning to NRP is critical to ensure the resuscitation protocol aligns with best practices for neonatal care. This approach highlights the importance of a seamless shift between protocols in settings where neonates may first present to non-specialized care providers, ensuring optimal outcomes through a multi-faceted, protocol-driven approach.

Scenario 2: A term neonate born at 38 weeks' gestation is referred to a tertiary care NICU (no in-house delivery services) shortly after birth due to respiratory distress. The baby was initially breathing but soon experienced significant oxygen desaturation, bradycardia with HR <80bpm, delayed capillary refill, and difficulty maintaining adequate respiratory efforts.

The most common etiology of respiratory distress in a term neonate shortly after birth may be due to meconium aspiration, retained fetal lung fluid, or pneumonia. NRP emphasizes gentle ventilation methods to avoid injury to the fragile neonatal lungs. In a tertiary referral center without delivery services, NRP and PALS may be used per the unit’s protocol. However, PALS providers may be more aggressive in ventilation and airway management compared to the more cautious practices of NRP for neonates.

In this situation, the use of NRP would be more suitable because it is specifically designed for neonates, considering their unique physiological needs, including more delicate respiratory management and preventing further complications, such as hypoxia or hyperoxia.

Given the absence of clear evidence favoring one protocol over another in this scenario, the initial use of PALS is understandable. However, as soon as a neonatal provider is available, transitioning to NRP would ensure that the resuscitation aligns with best practices tailored to the newborn’s specific physiological characteristics. Neonatal providers trained in NRP are well-equipped to manage neonates’ respiratory distress and bradycardia, focusing on ventilation, oxygenation, and stabilization through neonatal-specific strategies.

While there is no definitive evidence to refute one protocol over the other, available guidelines suggest that transitioning to NRP upon the arrival of a neonatal specialist is the most appropriate approach for managing respiratory distress and bradycardia in neonates, ensuring the best outcomes for the newborn.

Scenario 3: A preterm neonate in the NICU, a former 29-weeker, now corrected to 40 weeks postmenstrual age, is currently on a 2 L nasal cannula with supplemental oxygen of 25%. She has a severe apnea/bradycardia/desaturation episode about 2 hours after a feed. A travel nurse caring for this infant starts resuscitation as per the PALS algorithm.

For a preterm infant experiencing a severe apneic episode, bradycardia, and desaturation, the first-line management typically includes tactile stimulation, supplemental oxygen, and airway clearance, followed by positive pressure ventilation if necessary. This approach is effective in resolving mild to moderate apneic events. If the infant remains unresponsive to these measures, resuscitation efforts are escalated, often involving positive pressure ventilation or even intubation, depending on the severity of the situation.

However, airway management and ventilation are particularly important in neonates, especially preterm infants. As this infant is not intubated, focus should be on appropriate ventilation to restore the heart rate and improve oxygenation. NRP provides clear guidance in this area, emphasizing proper airway management and ventilation to correct bradycardia and desaturation, which are common in preterm infants experiencing apnea of prematurity or related respiratory complications.

In this scenario, the travel nurse initiated PALS guidelines. While the PALS algorithm is often used for resuscitation in older infants and children, its application in this scenario could still be justified, particularly in an environment where a provider not trained in NRP was at the bedside. However, NRP would be more appropriate for this infant to address the underlying causes of apnea and bradycardia.

Since the infant is on supplemental oxygen and experiencing an apnea/bradycardia/desaturation event, the initial steps, such as ensuring airway patency and applying positive pressure ventilation, would fall within the scope of NRP guidelines. While the PALS algorithm may still provide a framework for resuscitation, a provider trained in PALS may start compressions before optimizing ventilation.

If this infant was being managed in a pediatric floor for bronchopulmonary dysplasia (BPD) and experienced a similar episode, the application of PALS could be considered appropriate, as PALS is designed for managing such events in older infants and children. However, no clear evidence exists to refute either approach, and NRP and PALS could be utilized depending on the circumstances. Transitioning to NRP when a neonatal specialist is available would be ideal to ensure the most appropriate resuscitation strategy is employed. This scenario underscores the importance of tailored resuscitation protocols in the NICU, particularly for preterm infants who may have specific respiratory needs.

To summarize, these scenarios demonstrate the complexities and challenges of neonatal resuscitation, particularly in settings where neonatal providers may not be immediately available. While both NRP and PALS have their merits, the protocols are designed for different patient populations, and the transition from PALS to NRP is crucial when caring for neonates. In each scenario, the initial use of PALS may be justifiable, but as soon as a neonatal provider is available, transitioning to NRP is essential for ensuring that resuscitation is aligned with the best practices for neonates, particularly for their unique physiological needs and challenges. On pediatric floors and in pediatric intensive care units, some infants may be resuscitated using PALS; however, currently a dilemma exists in choosing one over the other.

#### Guidelines for infant (age <12 months) resuscitation

NRP and PALS are the two guidelines recommended by the American Heart Association (AHA) that are widely used in the resuscitation of infants [12, 13]. A national survey analyzed the resuscitation practices of the US healthcare providers (*n* = 152) in three different settings, including 118 NICUs, 19 pediatric intensive care units (PICUs), and 15 cardiac intensive care units (CICUs) [14]. The survey results showed that *neonatal providers* preferred neonatal guidelines in infants <28 days and pediatric guidelines in infants beyond 28 days with a primary cardiac etiology for the arrest. In contrast, the providers in the PICU and CICU preferred pediatric guidelines for any infant after birth, irrespective of the age or etiology of the cardiac arrest. The provider’s preference determined the application of either NRP or PALS based on similar foundational principles of resuscitation.

The fundamental differences between NRP vs. PALS were discussed in a recent report published collaboratively on behalf of the AHA Emergency Cardiovascular Care Committee and the American Academy of Pediatrics (AAP) [11]. The emphasis is on adequate ventilation in the NRP guidelines and CC in the PALS guidelines (Table 1). The NRP recommends 3 CC to 1 breath with a pause for the breaths, including in the presence of an advanced airway [15]. There are 120 events in a minute (90 CC and 30 breaths). The PALS guidelines recommends for one rescuer include 30 CC to 2 breaths, or 15 CC to 2 breaths for two-rescuer resuscitations with no advanced airway [13]. Two rescuers with an advanced airway include continuous CC with one breath every 2-3 seconds, which adds up to 100-120 CC to 20 -30 breaths in a minute [16]. Epinephrine is the only medication recommended in NRP with blood transfusion and a normal saline bolus during special circumstances [17]. PALS recommends multiple medications, cardioversion, and defibrillation based on the various rhythm abnormalities through algorithms relatively more complex than NRP [18].

**Table 1:** Summarizing the perspective.

Guidelines	NRP (Neonatal Resuscitation Program)	PALS (Pediatric Advanced Life Support)
**Population**	Newborns (0–28 days old)	Infants and children (infants, children, and adolescents up to 18 years of age, excluding newborns)
**Common etiology of arrest**	Perinatal asphyxia, respiratory failure	Respiratory failure, shock, arrhythmias
**Primary Focus**	Ventilation, oxygenation, thermoregulation, and heart rate support	Cardiac compressions, airway management, and resuscitation
**Initial Resuscitation**	Emphasizes positive pressure ventilation, ventilation corrective steps, and chest compressions	Emphasizes chest compressions, airway management, and advanced interventions
**CPR Sequence**	3:1 compression to ventilation ratio. Pulse checks every minute after effective chest compressions	In one-rescuer CPR, the compression-to-ventilation ratio is 30:2; in two-rescuer CPR, it is 15:2. Pulse checks every 2 minutes.
**Medications**	Epinephrine (first line), normal saline	Epinephrine, amiodarone, and other drugs depending on the situation
**Provider Level**	Trained neonatal resuscitation providers, typically neonatologists or neonatal nurses who are at the forefront in the delivery room and in the NICU	Trained pediatric care providers, including pediatricians, paramedics, and emergency medical technicians
**Special Considerations**	Focus on thermal regulation, avoiding hypothermia, and specific neonatal anatomy	Focus on managing various pediatric medical emergencies, including arrhythmias and trauma

#### Identification of pulse – Salient points of divergence

NRP does not distinguish the resuscitation of infants with severe bradycardia (HR < 60 bpm), asystole, and pulseless electrical activity (PEA). NRP has a common approach toward different etiologies of cardiac arrest. PALS recommendations for severe bradycardia with a pulse are distinct from asystole/PEA. The choice of these approaches depends on identifying the brachial pulse in the infants. The identification of pulses in severe bradycardia determines whether the initial resuscitative efforts are being targeted toward optimizing oxygenation and ventilation versus analyzing the rhythm with an automated external defibrillator (AED) and starting high-quality CPR. NRP recommends epinephrine, preferably intravascular if the HR is <60 bpm despite 1 min of coordinated effective PPV + CC. In a similar situation, pediatric guidelines recommend epinephrine and atropine.

## What about the Epinephrine dose, and how do we handle it?

Based on all the studies in newborns, the first dose of epinephrine (0.02 mg/kg) was administered 5 minutes from the onset of resuscitation if ROSC was not achieved [19]. NRP suggests using a higher initial dose of epinephrine (0.02 mg/kg) within a range of 0.01 mg/kg – 0.03 mg/kg for educational efficiency and to prevent dosing preparation errors [20]. However, the preclinical data suggest that a higher initial dose of epinephrine within the NRP recommended range may be efficacious [21–24]. The dose of epinephrine recommended by PALS is 0.01 mg/kg per dose, which falls within the lower dose range recommended by NRP [25].

## How can we resuscitate a 6-month-old in the NICU if we don’t have equipoise?

NRP defines adequate ventilation as PPV with visible chest rise for 30 seconds before initiation of CC and recommends placing an advanced airway, such as an endotracheal tube (ETT) or laryngeal mask (LM), before initiating CC. PALS recommends an advanced airway after analyzing the rhythm, administering epinephrine, and performing high-quality CPR for 2 minutes. The 2019 PALS update recommended that bag-mask ventilation (BMV) is reasonable compared with advanced airway interventions (endotracheal intubation or supraglottic airway) in managing children during cardiac arrest in the out-of-hospital setting [16]. However, there is insufficient evidence for or against using advanced airways in IHCA in pediatric patients. A recent multicenter, international registry study including 5453 children with difficult intubation found that BMV was difficult in 9% of the study population [26]. Age <12 months and weight for age (<5th percentile and >95th percentile) were associated with an increased risk of difficult BMV. Prematurity is a leading cause of IHCA in early infancy [8]. High-risk premature infants with exposure to prolonged invasive mechanical ventilation and underlying chronic lung disease are at risk of airway damage leading to acquired airway obstructive lesions such as subglottic stenosis and subglottic cysts, putting them at higher risk for difficult BMV [27]. Obtaining an advanced airway, such as ETT & LM, as recommended, is also essential.

## Optimizing ventilation strategies in infant resuscitation: Point of convergence

Hypoxia, secondary to respiratory failure, leading to bradycardia, is the most common precipitating cause of IHCA in infants, which both NRP and PALS acknowledge in their guidelines [16, 28, 29]. The focus of the initial steps of resuscitation should be to optimize oxygenation and ventilation. NRP recommends rescue breathing at 40–60 per minute before starting CC and 30 breaths per minute during coordinated CC. This recommendation is based on the observational data of the average respiratory rates among infants. PALS recommends rescue breathing at a rate of 20 -30 per minute before and during CC [13]. This recent recommendation change was provided for the ease of training with limited evidence [30]. PALS has acknowledged the critical knowledge gap due to insufficient pediatric data regarding the optimal ventilation rate during CPR in patients with or without an advanced airway and whether the ventilation rate is age-dependent. A systematic review of observational studies found that the respiratory rates declined from a median of 44 per minute at birth to 26 per minute at the end of 2 years, which continued to decline until early adolescence [31]. Given the differences in respiratory rates and the algorithm, is it reasonable to resuscitate all infants per NRP during an IHCA, especially in the NICU?

## Chest compressions in infant CPR – Double-Edged Sword?

NRP has recommended electrocardiographic (ECG) monitoring of the heart rate in infants undergoing PPV at birth since 2015. A single-center study in the US compared the impact of this practice-changing recommendation using a prospective and a retrospective cohort [32]. The authors found that the use of ECG in the delivery room caused a 3.6-fold increase in the rate of CC. A retrospective observational study of neonatal CPR at birth (*n* = 1153) using the AHA Get With The Guidelines-Resuscitation registry found that CC were started in the first minute of life in 76% of CPR events, and 79% of infants received CC without endotracheal intubation [33]. A similar registry-based retrospective study analyzed the prevalence and predictors of survival of IHCA in 5592 pediatric patients (age >30 days of life - <18 years of age) who progressed from bradycardia to pulselessness. The study reported that 50% of the cohort had bradycardia with poor perfusion at the start of CC, with approximately 30% of those patients progressing to pulselessness despite CPR [34]. Progression to pulselessness despite CPR resulted in decreased survival compared to pulseless cardiac arrest, with an inverse relationship to the time to pulselessness. The reasons for the progression and poor survival of patients with bradycardia with pulse to pulselessness despite CPR are unknown.

CC in NRP and PALS differ primarily due to the age of the patient. In NRP, CC for newborns is typically performed using the 2-thumb technique or encircling hands for a depth of approximately one-third of the anterior-posterior diameter of the chest. The compression-to-ventilation ratio is usually 3:1, aiming to give 90 compressions and 30 ventilations per minute, accounting for 120 events per minute. In PALS, CC for children is performed with either a one- or two-hand technique, depending on size, and for infants, the two-finger or two-thumb technique is used. The compression depth for infants is about 1.5 inches (4 cm), and for children, it is about 2 inches (5 cm), with a rate of 100–120 compressions per minute with an advanced airway in place. The compression-to-ventilation ratio is 30:2 for single rescuers and 15:2 for two rescuers.

## Adoption of PALS guidelines in the NICU

Arrhythmias - NRP recommends using neonatal guidelines during the immediate newborn period and the initial hospitalization after birth [35, 36]. In other words, NRP recommends the use of a 3:1 compression-to-ventilation ratio in infant CPR occurring in the NICU unless there is a strong suspicion of primary cardiac arrhythmia or dyselectrolytemia being the cause of the IHCA. PALS guidelines include specific recommendations to resuscitate infants experiencing IHCA secondary to etiologies such as cardiac arrhythmias and septic shock that may be relevant in a significant proportion of infants in the NICU [13]. Such scenarios are prevalent in the quaternary NICUs that tend to care for complex infants after birth until they are several weeks or months old [37]. Prompt use of an AED and medications such as amiodarone that are not included in the NRP framework could be lifesaving in select situations [38].

Evidence is lacking to identify a specific age cutoff after birth to transition from NRP to PALS guidelines for infants with IHCA. Etiology-based selection of patients for resuscitation using PALS guidelines for IHCA occurring in the NICU is a reasonable approach. A quaternary NICU in the US has reported the successful implementation of a quality improvement initiative to transition from NRP to a combined NRP-PALS model based on the post-menstrual age and etiology [39]. The infants who were ≥ 44 weeks PMA at the time of the IHCA event or post-cardiac surgery for an etiology other than patent ductus arteriosus or diagnosis of cardiac arrhythmia during the event underwent resuscitation based on PALS guidelines. There are wide variations in the choice of guidelines by various NICUs [40]. The choice between NRP and PALS is sometimes determined by the patient characteristics specific to the unit. For example, a quaternary NICU in the US that exclusively treats outborn infants ranging from 0-9 months old, transferred into the unit, uses PALS guidelines for all infantile CPR [41].

## Challenges surrounding infant CPR

The recent joint report by the AHA Emergency Cardiovascular Care Committee and the AAP described the problems prevalent in understanding the effectiveness of NRP vs. PALS guidelines in resuscitation of infants with IHCA [11]. The report highlighted the lack of good-quality evidence, limiting the ability to make recommendations for the transition from NRP to PALS. The primary hurdle in generating evidence to resolve this knowledge gap is the presence of various pediatric subspecialties (NICU, PICU, CICU, pediatric emergency medicine) that use different sets of evidence to create guidelines for infant CPR. NRP has to retain its focus on delivery room resuscitation, while PALS has to address a wide range of etiologies of cardiac arrest, such as drowning, trauma, and toxicology. This broad range of other etiologies is not likely to be relevant to IHCA in infants during their birth hospitalization in the NICU and who are subsequently transferred to the PICU for a continuum of care. A well-designed randomized comparative effectiveness trial could give us definitive answers to guide the practice; however, informed parental consent for such high acuity, high-stakes intervention could be challenging [42]. Maintenance of skills, fluency, and efficiency in 2 different guidelines applied in low-frequency acute events among healthcare providers could be challenging even in large quaternary intensive care units [43].

## Cost factor while training patient care providers in both NRP and PALS

The cost of NRP course can vary depending on the care providers and the course components, ranging from $45 to $280 USD. This does not include the additional cost of the NRP book. The NRP course typically lasts one day. In contrast, the PALS course requires a Basic Life Support (BLS) certification as a prerequisite and spans two days, with costs ranging from $150 to $250 USD, with an additional cost for the textbook.

The costs associated with maintaining these training programs can also vary based on the location of the providers (hospital versus outpatient settings). Training NICU staff in both NRP and PALS may incur higher costs compared to training ER or PICU providers, who typically only require PALS training. Additionally, hidden costs in hospital settings include the regular wages paid to staff during training hours.

## Conclusion

While resuscitating a neonate in the NICU, there is currently no definitive evidence to support the superiority of either the NRP or PALS protocol. Both protocols provide valuable frameworks for managing emergencies, yet studies comparing their outcomes and effectiveness in this specific setting remain limited. While NRP is designed specifically for neonatal resuscitation, and PALS offers a more generalized approach to pediatric cardiac care, there is a lack of consensus regarding which protocol is more beneficial in the NICU. Following AHA recommendations, institutions should consider developing policies tailored to meet their respective populations’ unique patient care needs.

NRP guidelines could be enhanced by incorporating specific therapies for cardiac arrhythmias and recommendations for infants with congenital cardiac anomalies or primary cardiac etiologies of cardiac arrest, which are currently under development [44]. This would allow providers using NRP to better refine their resuscitation strategies for less common clinical scenarios [45].

Additionally, PALS can help highlight key differences in infant resuscitation protocols, particularly by updating the cardiac arrest algorithm to address resuscitation strategies for infants with severe bradycardia who still have a pulse [46]. Further research is needed to evaluate the outcomes associated with each protocol in neonatal care and to guide evidence-based decisions on the most appropriate training and implementation for NICU providers.

## Data Availability

As cited.

## References

[1] Moshiro R, Mdoe P, Perlman JM. A global view of neonatal asphyxia and resuscitation. Front Pediatr. 2019;7:489. 31850287 10.3389/fped.2019.00489 PMC6902004

[2] Burchfield DJ. Newborn resuscitation. Clin Pediatr Emerg Med. 2001;2:119–23.

[3] Rainaldi MA, Perlman JM. Pathophysiology of Birth Asphyxia. Clin Perinatol. 2016;43:409–22. 27524444 10.1016/j.clp.2016.04.002

[4] Foglia EE, Te Pas AB. Effective ventilation: The most critical intervention for successful delivery room resuscitation. Semin Fetal Neonatal Med. 2018;23:340–6. 29705089 10.1016/j.siny.2018.04.001 PMC6288818

[5] Weiner GM, Zaichkin J, Bloom RS. Textbook of neonatal resuscitation. 8th edition. ed. Itasca, IL: American Academy of Pediatrics; 2021.

[6] Idris AH, Guffey D, Pepe PE, Brown SP, Brooks SC, Callaway CW, et al. Chest compression rates and survival following out-of-hospital cardiac arrest. Crit Care Med. 2015;43:840–8. 25565457 10.1097/CCM.0000000000000824

[7] Gupta P, Yan K, Chow V, Dao DT, Gossett JM, Leong K, et al. Variability of characteristics and outcomes following cardiopulmonary resuscitation events in diverse ICU settings in a single, tertiary care children’s hospital*. Pediatr Crit Care Med. 2014;15:e128–41. 24413318 10.1097/PCC.0000000000000067

[8] Foglia EE, Langeveld R, Heimall L, Deveney A, Ades A, Jensen EA, et al. Incidence, characteristics, and survival following cardiopulmonary resuscitation in the quaternary neonatal intensive care unit. Resuscitation. 2017;110:32–6. 27984153 10.1016/j.resuscitation.2016.10.012 PMC5193233

[9] Ahmad KA, Velasquez SG, Kohlleppel KL, Henderson CL, Stine CN, LeVan JM, et al. The characteristics and outcomes of cardiopulmonary resuscitation within the neonatal intensive care unit based on gestational age and unit level of care. Am J Perinatol. 2020;37:1455–61. 31365927 10.1055/s-0039-1693990

[10] Ali N, Lam T, Gray MM, Clausen D, Riley M, Grover TR, et al. Cardiopulmonary resuscitation in quaternary neonatal intensive care units: a multicenter study. Resuscitation. 2021;159:77–84. 33359416 10.1016/j.resuscitation.2020.12.010

[11] Sawyer T, McBride ME, Ades A, Kapadia VS, Leone TA, Lakshminrusimha S, et al. Considerations on the use of neonatal and pediatric resuscitation guidelines for hospitalized neonates and infants: on behalf of the American Heart Association Emergency Cardiovascular Care Committee and the American Academy of Pediatrics. Pediatrics. 2024;153:7. 10.1542/peds.2023-064681 38105696

[12] Aziz K, Lee HC, Escobedo MB, Hoover AV, Kamath-Rayne BD, Kapadia VS, et al. Part 5: Neonatal Resuscitation: 2020 American Heart Association Guidelines for Cardiopulmonary Resuscitation and Emergency Cardiovascular Care. Circulation. 2020;142:S524–50. 33081528 10.1161/CIR.0000000000000902

[13] Topjian AA, Raymond TT, Atkins D, Chan M, Duff JP, Joyner BL Jr., et al. Part 4: Pediatric Basic and Advanced Life Support: 2020 American Heart Association Guidelines for Cardiopulmonary Resuscitation and Emergency Cardiovascular Care. Circulation. 2020;142:S469–s523. 33081526 10.1161/CIR.0000000000000901

[14] Ali N, Sawyer T, Barry J, Grover T, Ades A. Resuscitation practices for infants in the NICU, PICU and CICU: results of a national survey. J Perinatol: J Calif Perinat Assoc. 2017;37:172–6. 10.1038/jp.2016.193 27787506

[15] Textbook of Neonatal Resuscitation: American Academy of Pediatrics. Available from: 10.1542/9781610025256.

[16] Duff JP, Topjian AA, Berg MD, Chan M, Haskell SE, Joyner BL Jr., et al. 2019 American Heart Association Focused Update on Pediatric Advanced Life Support: An Update to the American Heart Association Guidelines for Cardiopulmonary Resuscitation and Emergency Cardiovascular Care. Circulation. 2019;140:e904–e14. 31722551 10.1161/CIR.0000000000000731

[17] Vali P, Sankaran D, Rawat M, Berkelhamer S, Lakshminrusimha S. Epinephrine in neonatal resuscitation. Children. 2019;6. 10.3390/children6040051 PMC6518253 30987062

[18] Ross CE, Moskowitz A, Grossestreuer AV, Holmberg MJ, Andersen LW, Yankama TT, et al. Trends over time in drug administration during pediatric in-hospital cardiac arrest in the United States. Resuscitation. 2021;158:243–52. 33147522 10.1016/j.resuscitation.2020.09.040 PMC7851749

[19] Barber CA, Wyckoff MH. Use and efficacy of endotracheal versus intravenous epinephrine during neonatal cardiopulmonary resuscitation in the delivery room. Pediatrics. 2006;118:1028–34. 16950994 10.1542/peds.2006-0416

[20] Wyckoff M, Wyllie J, Aziz K, de Almeida M, Fabres J, Fawke J, et al. Neonatal Life Support: 2020 International Consensus on Cardiopulmonary Resuscitation and Emergency Cardiovascular Care Science With Treatment Recommendations. Circulation. 2020;142:S204. 10.1161/CIR.0000000000000895 33084392

[21] Vali P, Chandrasekharan P, Rawat M, Gugino S, Koenigsknecht C, Helman J, et al. Evaluation of timing and route of epinephrine in a neonatal model of asphyxial arrest. J Am Heart Assoc. 2017;6:9–10. 10.1161/JAHA.116.004402 PMC5523751 28214793

[22] Vali P, Chandrasekharan P, Rawat M, Gugino S, Koenigsknecht C, Helman J, et al. Continuous chest compressions during sustained inflations in a perinatal asphyxial cardiac arrest Lamb Model. Pediatr Crit Care Med. 2017;18:e370–e7. 28661972 10.1097/PCC.0000000000001248 PMC5552419

[23] Sankaran D, Chandrasekharan P, Gugino S, Koenigsknecht C, Helman J, Nair J, et al. Randomised trial of epinephrine dose and flush volume in term newborn lambs. Arch Dis Child - Fetal Neonatal Ed. 2021;106:578–83. 33687959 10.1136/archdischild-2020-321034 PMC8543198

[24] Songstad N, Klingenberg C, McGillick E, Polglase G, Zahra V, Schmölzer G, et al. Efficacy of Intravenous, Endotracheal, or Nasal Adrenaline Administration During Resuscitation of Near-Term Asphyxiated Lambs. Front Pediatr. 2020;8:8–9. 10.3389/fped.2020.00262 PMC7282342 32582589

[25] Duff JP, Topjian A, Berg MD, Chan M, Haskell SE, Joyner BL, et al. 2018 American Heart Association Focused Update on Pediatric Advanced Life Support: An Update to the American Heart Association Guidelines for Cardiopulmonary Resuscitation and Emergency Cardiovascular Care. Circulation. 2018;138:e731–e9. 30571264 10.1161/CIR.0000000000000612

[26] Garcia-Marcinkiewicz AG, Lee LK, Haydar B, Fiadjoe JE, Matava CT, Kovatsis PG, et al. Difficult or impossible facemask ventilation in children with difficult tracheal intubation: a retrospective analysis of the PeDI registry. Br J Anaesth. 2023;131:178–87. 37076335 10.1016/j.bja.2023.02.035

[27] Zhang H, Zhang J, Zhao S. Airway damage of prematurity: The impact of prolonged intubation, ventilation, and chronic lung disease. Semin Fetal Neonatal Med. 2016;21:246–53. 27129915 10.1016/j.siny.2016.04.001

[28] Moler FW, Silverstein FS, Holubkov R, Slomine BS, Christensen JR, Nadkarni VM, et al. Therapeutic hypothermia after out-of-hospital cardiac arrest in children. N. Engl J Med. 2015;372:1898–908. 25913022 10.1056/NEJMoa1411480 PMC4470472

[29] Morgan RW, Kirschen MP, Kilbaugh TJ, Sutton RM, Topjian AA. Pediatric in-hospital cardiac arrest and cardiopulmonary resuscitation in the United States: A review. JAMA Pediatr. 2021;175:293–302. 33226408 10.1001/jamapediatrics.2020.5039 PMC8787313

[30] Sutton RM, Reeder RW, Landis WP, Meert KL, Yates AR, Morgan RW, et al. Ventilation rates and pediatric in-hospital cardiac arrest survival outcomes. Crit Care Med. 2019;47:1627–36. 31369424 10.1097/CCM.0000000000003898 PMC7898415

[31] Fleming S, Thompson M, Stevens R, Heneghan C, Plüddemann A, Maconochie I, et al. Normal ranges of heart rate and respiratory rate in children from birth to 18 years of age: a systematic review of observational studies. Lancet. 2011;377:1011–8. 21411136 10.1016/S0140-6736(10)62226-X PMC3789232

[32] Shah BA, Wlodaver AG, Escobedo MB, Ahmed ST, Blunt MH, Anderson MP, et al. Impact of electronic cardiac (ECG) monitoring on delivery room resuscitation and neonatal outcomes. Resuscitation. 2019;143:10–6. 31394156 10.1016/j.resuscitation.2019.07.031

[33] Halling C, Raymond T, Brown LS, Ades A, Foglia EE, Allen E, et al. Neonatal delivery room CPR: An analysis of the Get with the Guidelines®-Resuscitation Registry. Resuscitation. 2021;158:236–42. 33080368 10.1016/j.resuscitation.2020.10.007

[34] Khera R, Tang Y, Girotra S, Nadkarni VM, Link MS, Raymond TT, et al. Pulselessness after initiation of cardiopulmonary resuscitation for bradycardia in hospitalized children. Circulation. 2019;140:370–8. 31006260 10.1161/CIRCULATIONAHA.118.039048 PMC6663562

[35] Textbook of Neonatal Resuscitation. Weiner GM, Zaichkin J, editors: American Academy of Pediatrics; 2021.

[36] Wyckoff MH, Sawyer T, Lakshminrusimha S, Collins A, Ohls RK, Leone TA. Resuscitation 2020: Proceedings From the NeoHeart 2020 International Conference. World J Pediatr Congenit heart Surg. 2022;13:77–88. 34919486 10.1177/21501351211038835

[37] Morrow CB, McGrath-Morrow SA, Collaco JM. Predictors of length of stay for initial hospitalization in infants with bronchopulmonary dysplasia. J Perinatol: J Calif Perinat Assoc. 2018;38:1258–65. 10.1038/s41372-018-0142-7 PMC6195847 29880793

[38] Rossano JW, Jones WE, Lerakis S, Millin MG, Nemeth I, Cassan P, et al. The use of automated external defibrillators in infants: A Report From the American Red Cross Scientific Advisory Council. Pediatr Emerg Care. 2015;31:526–30. 26148104 10.1097/PEC.0000000000000490

[39] Harer MW, Konkol LJ, Limjoco JJ. Transitioning from NRP to a combined PALS-NRP resuscitation model at a level IV NICU. J Perinatol: J Calif Perinat Assoc. 2022;42:1533–4. 10.1038/s41372-022-01416-3 PMC9617750 35610362

[40] Gover A, Levy PT, Zaltsberg-Barak T, Rotschild A, Molad M, Lavie-Nevo K, et al. Neonatal resuscitation in the NICU; Challenges beyond NRP. Acta Paediatr. 2021;110:3269–71. 34347316 10.1111/apa.16057

[41] Raju RM, Leeman KT. Pediatric advanced life support in a neonatal context. Neoreviews. 2022;23:e359–e62. 35490183 10.1542/neo.23-5-e359

[42] Nichol G, Huszti E, Rokosh J, Dumbrell A, McGowan J, Becker L. Impact of informed consent requirements on cardiac arrest research in the United States: exception from consent or from research? Resuscitation. 2004;62:3–23. 15246579 10.1016/j.resuscitation.2004.02.013

[43] Binkhorst M, van der Aar IM, Linders M, van Heijst AFJ, de Boode WP, Draaisma JMT, et al. Training practices in neonatal and paediatric life support: A survey among healthcare professionals working in paediatrics. Resuscitation. 2021;5:100063. 10.1016/j.resplu.2020.100063 PMC8244515 34223335

[44] Jakubow A. Shock unresponsive to Neonatal Resuscitation Program interventions. Can Fam Physician. 2022;68:438–40. 35701202 10.46747/cfp.6806438 PMC9197293

[45] Kalaniti K, Schmölzer GM, McNamara PJ. Neonatal resuscitation beyond the delivery room - does one protocol fit all? Acta Paediatr. 2015;104:971–3. 26174225 10.1111/apa.13116

[46] Baruteau AE, Perry JC, Sanatani S, Horie M, Dubin AM. Evaluation and management of bradycardia in neonates and children. Eur J Pediatr. 2016;175:151–61. 26780751 10.1007/s00431-015-2689-z

